# Supplementary material for: Prevalence and Relationship of Rest Tremor and Action Tremor in Parkinson’s Disease
Source: Tremor Other Hyperkinet Mov (N Y). 2020 Dec 23;10:58. doi: 10.5334/tohm.552 (PMC7757606; doi:10.5334/tohm.552)
Supplement: Supplementary Table 1. — Multivariate logistic regression analysis of prevalence of action tremor and rest tremor, adjusting for age, gender, and disease duration across the baseline data of each cohort. [file tohm-10-1-552-s1.pdf]

Supplementary Table 1. Multivariate logistic regression analysis of prevalence of action tremor and rest tremor, adjusting for age, gender, and disease duration across the baseline data of each cohort.

Variable	PPMI	BioFIND	PDBP
Rest tremor	1.72 (1.09 – 2.73)*	3.15 (1.30 – 7.62)*	3.77 (2.79 – 5.10)**
Male gender	1.69 (1.09 – 2.61)*	1.87 (0.80 – 4.36)	1.87 (1.05 – 1.94)*
Disease duration (months)	1.00 (0.97 – 1.02)	0.99 (0.98 – 1.00)	NA
Age	0.98 (0.96 – 1.00)	0.94 (0.88 – 1.01)	1.01 (0.99 – 1.03)

Table 1 Legend: Parkinson Progression Marker Initiative (PPMI), The Fox Investigation for New Discovery of Biomarkers (BioFIND) and Parkinson's Disease Biomarkers Program (PDBP). NA - Disease duration variable was not available in the PDBP database. Data are reported as OR (95% CI). *p<0.05, **p<0.001
